# Efficient Test and Visualization of Multi-Set Intersections

**DOI:** 10.1038/srep16923

**Published:** 2015-11-25

**Authors:** Minghui Wang, Yongzhong Zhao, Bin Zhang

**Affiliations:** 1 Department of Genetics and Genomic Sciences, Icahn Institute of Genomics and Multiscale Biology, Icahn School of Medicine at Mount Sinai, 1470 Madison Avenue, NY 10029, USA

## Abstract

Identification of sets of objects with shared features is a common operation in all disciplines. Analysis of intersections among multiple sets is fundamental for in-depth understanding of their complex relationships. However, so far no method has been developed to assess statistical significance of intersections among three or more sets. Moreover, the state-of-the-art approaches for visualization of multi-set intersections are not scalable. Here, we first developed a theoretical framework for computing the statistical distributions of multi-set intersections based upon combinatorial theory, and then accordingly designed a procedure to efficiently calculate the exact probabilities of multi-set intersections. We further developed multiple efficient and scalable techniques to visualize multi-set intersections and the corresponding intersection statistics. We implemented both the theoretical framework and the visualization techniques in a unified R software package, *SuperExactTest*. We demonstrated the utility of *SuperExactTest* through an intensive simulation study and a comprehensive analysis of seven independently curated cancer gene sets as well as six disease or trait associated gene sets identified by genome-wide association studies. We expect *SuperExactTest* developed by this study will have a broad range of applications in scientific data analysis in many disciplines.

“Sets” are a commonly used concept in all disciplines. Classification of distinct objects into sets is a basic operation in analyzing and understanding the relationships of the objects. For example, in biology sciences, gene signatures, which are lists of genes of common expression patterns with respect to certain perturbations or phenotypes^1^,^2^, can be treated as sets; grouping genes into biologically meaningful gene sets facilitates our understanding of the genomes. While identification of sets from a population of objects is of primary interest in scientific data analysis, it is natural to study the relationships among multiple sets via measuring and visualizing their connections by intersecting them. Many similarity indices such as Sørensen coefficient^3^ and the Jaccard index^4^ have been proposed to measure the degree of commonalties and differences between two sets. Assuming independent sampling of a collection of objects into each set, the standard Fisher’s exact test (FET)^5^ or hypergeometric test^6^ can be employed to calculate the statistical significance of the observed overlap (i.e. intersection) between two sets. FET has been widely used in evaluating the enrichment of known functional pathways in predicted gene signatures^7^. When the intersection goes beyond two sets, computing the statistical distribution of the high-order intersections is not trivial. One solution is to perform repeated simulations^1^. However, the simulation analysis can only give rise to an approximate estimate and is computationally inefficient when the number of sets increases, particularly in cases in which the cardinality of a sample space is large but the expected overlap size is small. As the analysis of high-order relationships among multiple sets is fundamental for our in-depth understanding of their complex mechanistic interactions, there is an urgent need for developing robust, efficient and scalable algorithms to assess the significance of the intersections among a large number of sets.

Effective visualization of the comprehensive relationships among multiple sets is also of great interest and importance^8^. Venn diagrams have been the most popular way for illustrating the relationships between a very small number of sets, but are not feasible for more than five sets due to combinatorial explosion in the number of possible set intersections (2^*n*^ intersections for *n* sets). Although there is a plethora of methods and tools (e.g., VennMaster^9^,^10^, venneuler^11^ and UpSet^12^) to either axiomatically or heuristically resolve the issue of optimized visualization of multi-set intersections, a quantitative visualization of many complex relationships among multiple sets remains a challenge. For example, VennDiagram^13^, a popular Venn diagram plotting tool, can plot no more than five sets and thus has limited applications. It is even more challenging for VennDiagram to draw intersection areas proportional to their sizes. An alternative approach is to plot area-proportional Euler diagrams by using shapes like ellipses or rectangles to approximate the intersection sizes^14^. However, Euler diagram is only effective for a very small number of sets and is not scalable. Moreover, it is infeasible to present statistical significance of intersections in Venn or Euler diagram. Therefore, it is highly desirable to develop scalable visualization techniques for illustrating high-order relationships among multi-sets beyond Venn and Euler diagrams.

In this paper, we developed a theoretical framework to compute the statistical distributions of multi-set intersections based upon combinatorial theory and accordingly designed a procedure to efficiently calculate the exact probability of multi-set intersections. We further developed new scalable techniques for efficient visualization of multi-set intersections and intersection statistics. We implemented the framework and the visualization techniques in an R (http://www.r-project.org/) package, *SuperExactTest*. We demonstrated the utility of *SuperExactTest* through a comprehensive analysis of seven independently curated cancer gene signatures and six disease or trait associated gene sets identified by genome-wide association studies (GWAS).

## Results

### Implementation

We implemented the proposed multi-set intersection test algorithm in an R package *SuperExactTest*, which is available at CRAN (http://cran.r-project.org/).

The inputs for *SuperExactTest* include a list of vectors corresponding to multiple sets and the size of the background population from which the sets are sampled. The package enumerates the elements shared by every possible combination of the sets and then computes FE and the one-side probability for assessing statistical significance of each observed intersection. A generic summary function was implemented to tabulate all possible intersections, observed and expected sizes, FE values as well as probability values of significance tests.

### Effective Visualization of Multi-Set Intersections

**Figure 1:**
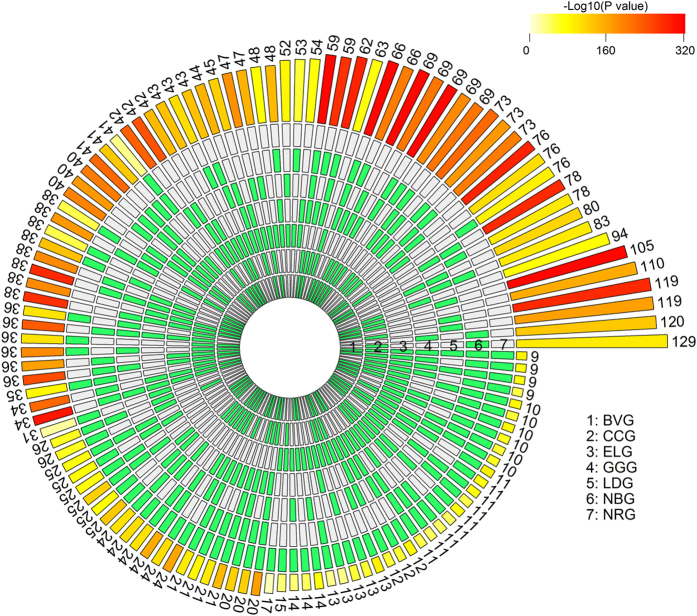
Visualization of the intersections amongst seven cancer gene sets. A circular plot illustrating all possible intersections and the corresponding statistics. The seven tracks in the middle represent the seven gene sets, with individual blocks showing “presence” (green) or “absence” (grey) of the gene sets in each intersection. The height of the bars in the outer layer is proportional to the intersection sizes, as indicated by the numbers on the top of the bars. The color intensity of the bars represents the P value significance of the intersections.

**Figure 2:**
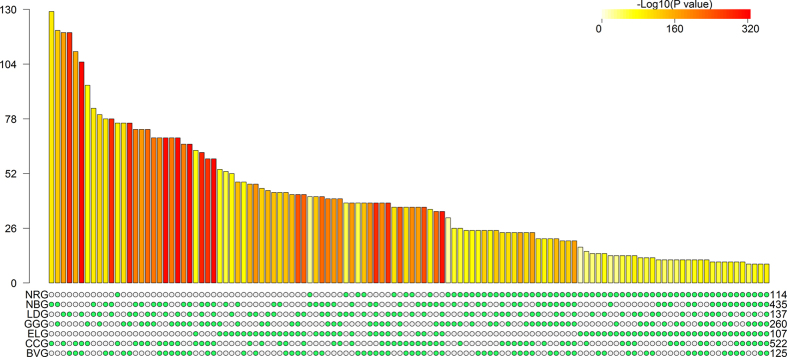
A bar chart illustrating all possible intersections among seven cancer gene sets in a matrix layout. The matrix of solid and empty circles at the bottom illustrates the “presence” (solid green) or “absence” (empty) of the gene sets in each intersection. The numbers to the right of the matrix are set sizes. The colored bars on the top of the matrix represent the intersection sizes with the color intensity showing the P value significance.

To facilitate the efficient identification and visualization of relations among a ‘large’ number of sets, we developed novel techniques for presenting multi-set intersections and significance tests. Instead of tweaking set bodies in a canvas, we proposed to organize all set intersections in a multi-layer circular^15^ layout or a two-dimensional matrix layout^12^ and then plot bars over the intersections (see Figs. 1 and 2 in the following sections). The bar height represents intersection size and the bar color intensity represents statistical significance (P values) of the intersections based on the statistical method we described in Methods. The colored bars can be sorted by the intersection size, set configuration or P value significance of FE. The matrix layout provides a straightforward presentation for a small number of sets while the circle layout is capable of plotting more sets with more properties (such as FE) by adding more tracks if needed. These visualization techniques provide an intuitive display of multi-set intersections in a relatively simple format and are capable of encoding more features than the traditional Venn and Euler diagrams. We note that at the time we prepared this manuscript, Lex and Gehlenborg^12^ introduced a web-based multi-set visualization tool which used a similar visualization strategy as our two-dimensional matrix layout. Both multi-set visualization approaches were implemented in the *SuperExactTest* R package so that users can perform the statistical test of multi-set intersections and visualize the results seamlessly.

### A Simulation Study of Intersection of Multiple Sets with Dependent Samples

The proposed method assumes that the sets under exact test are comprised of independent random samples from a population. However such an assumption may not be true in some applications. To explore whether the violation of the assumption of unbiased sampling could lead to some serious consequences, in particular inflated false positive rate in statistical tests, we designed a simulation study to assess the performance of the present method in analyzing the overlap among multiple sets derived from biased sampling. For simplicity, we considered a sampling scheme in which a portion of the elements in a population have a higher probability to be sampled than the rest. Specifically, we defined a weight *w* ( ≥ 1) as the ratio of the sampling probability for the group of the dependent elements to that for the rest elements: when *w* = 1, the sampling is unbiased, but when *w* > 1, some elements have a higher chance to be selected, mimicking the scenario that dependent elements are more likely to be sampled together than random. In each simulation, we sampled independently three sets of sizes 200, 300 and 400, from a population of size *n* and calculated the one-tailed P value significance of the overlap among the three sets. For each configuration of a population size *n* and a sampling weight *w*, we repeated the simulations 1,000 times and calculated the rate of false positives as the fraction of simulations with P value < 0.05 in the repeated simulations.

As shown in Table 1, the empirical false positive rate (FPR) is no larger than the expected level of 5% in the cases of unbiased sampling. Under biased sampling (*w* > 1), the FPR is well controlled in majority of the simulation settings. FPR is inflated only when the population size is small so that the preferential elements account for a relatively large fraction of the population. For example, when the preferential elements constitute 10% of the simulation populations of size 1,000, the empirical FPR departs from the expected level of 5% in cases of the sampling bias *w* ≥ 1.4. However, when the sample size is large and the fraction of preferential elements is less than 5%, the impact of biased sampling is negligible in the simulated populations. Our simulation approximates large scale genomic data analysis in that the background population consists of tens of thousands genes while gene sets under test, such as functional pathways, typically consist of tens or hundreds of members. The result demonstrates that the present method can be applied to cases when the sampling bias is absent or moderate, and when the population size is sufficiently large to mitigate the impact of sampling bias.

## Applications

### Consensus of cancer gene sets

Cancer genes are defined as the mutated genes that are causally implicated in oncogenesis^16^. After more than three decades of active searching, even with exploded systematic studies of the cancer genomes in recent years, the census of cancer genes remains an open question^17^. There are a number of cancer gene census sets available but the consistency across them has not been formally explored. To evaluate the extent to which existing cancer predisposition gene sets agree with each other and the connections between germline mutation of cancer predisposition genes and somatic cancer driver genes, we carried out a pan-cancer susceptible gene analysis using the *SuperExactTest* R package developed in this paper. We extensively searched the published large scale cancer genome studies and cancer review papers and derived seven core cancer gene census sets, namely NRG^18^, LDG^19^, GGG^17^, ELG^20^, CCG^16^, BVG^21^ and NBG^22^ based on the abbreviations of the corresponding author names (Table 2). NRG is a collection of germline mutated cancer predisposition genes while the rest are cancer predisposition gene censuses including both germline and somatic mutations. The sizes of these gene sets varies from 107 (ELG) to 522 (CCG). Assuming the cancer predisposition genes were randomly sampled from the population of 20,687 human genes, the intersections between any two cancer predisposition gene sets are highly significant (Bonferroni adjusted *P* values < 2.13 × 10^−18^, FEs > 11.75). Such significant overlaps among these cancer gene sets are not completely unexpected. As illustrated in Table S1 and Figs 1 and 2, we also calculated the intersections among three or more cancer predisposition gene sets. All the possible high-order intersections, i.e., the intersections across 3 or more sets, are very significant with adjusted *P* values < 6.05 × 10^−34^ and FEs > 529. All the seven sets share 9 genes including *ATM*, *CDKN2A*, *EGFR*, *NF1*, *PTEN*, *RUNX1*, *SMARCA4*, *STK11* and *TP53,* and this highest order intersection is highly significant, with FE = 5.7 × 10^10^ and adjusted *P* value = 4.3 × 10^−93^. This result is consistent with the well-recognized observations in large scale cancer genome discoveries that a few cancer genes are mutated in a high proportion of tumors of a given type (>20%) while most cancer gene mutations are less common (2–20%)^17^. All of these genes, except *EGFR*, which is a proto-oncogene characterized by gain of function, have been extensively studied as tumor suppressor genes characterized by loss of function. These genes play a variety of critical functional roles, such as genome stability maintenance and accurate cell cycle progression regulation (*PTEN* and *TP53*), metabolic regulation (*SMARCA4* and *STK11*), DNA damage sensor (*ATM*), as well as the cell cycle progression and proliferation (*CDKN2A*, *EGFR*, *NF1* and *RUNX1*). These are consensus pan-cancer driver genes, mutations in which have been found to be critical to the tumorigenesis in a number of malignancies.

**Figure 3:**
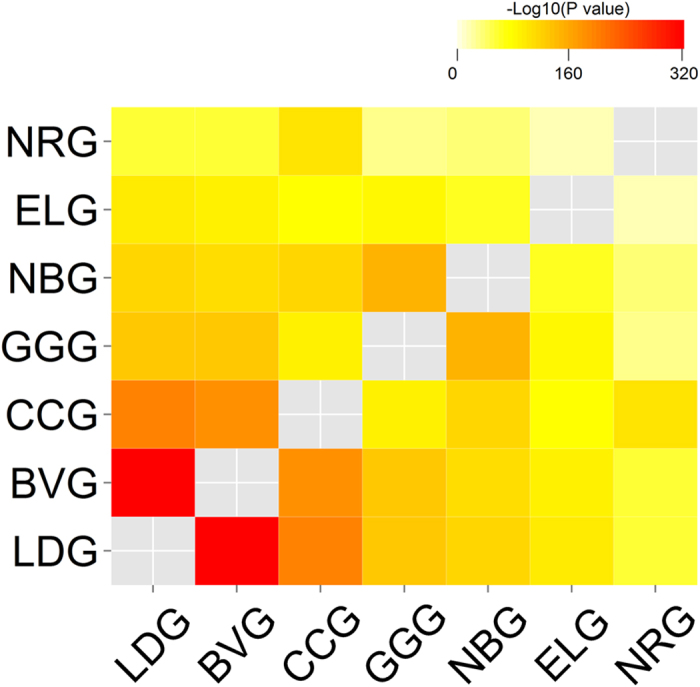
A heat-map illustrating the pairwise cancer gene set similarities as measured by the intersection analysis. The color intensity represents the *P* value significance of the intersection.

**Figure 4:**
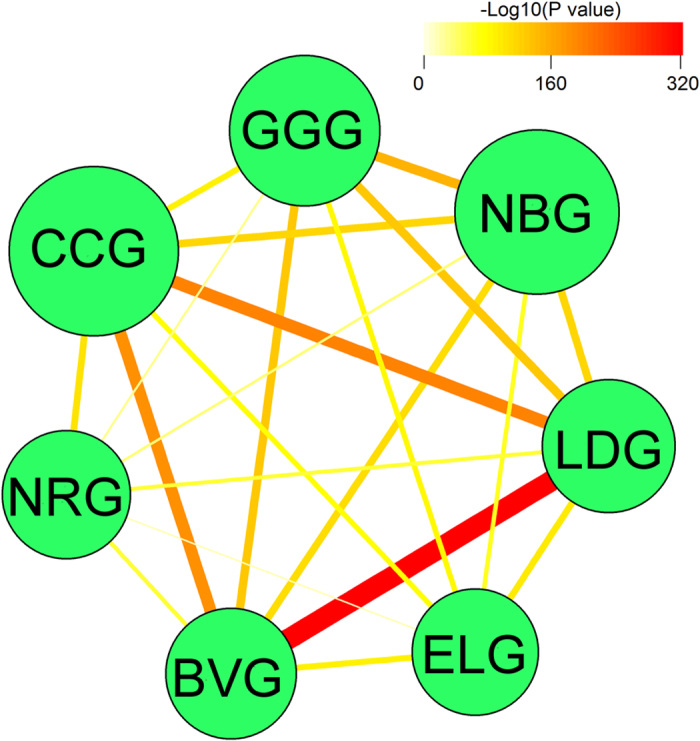
A network showing pairwise similarities among the seven cancer gene sets. The node size is proportional to the set size in log scale, while the edge width and edge color intensity represent the size and the *P* value significance of an intersection, respectively.

As the number of genes shared by all cancer predisposition gene sets is small, it is interesting to find out to which degree each set is consistent with the rest. We calculated the significance (*P* values) for all pairwise intersections and then for each set *i*, we rank-ordered the other six sets by the corresponding intersection *P* values. The ranking order of the consistency between a given set *i* and the remaining sets is denoted as 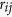 (for *j* = 1, …, 6). Following the metric for ranking causal regulatory genes^23^, we calculated a cumulative consistency score for each set *j* as 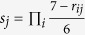. It is expected that a set consistently showing the strongest overlap with other sets will have the highest consistency score while the one with the weakest overlap with other sets will have the lowest consistency. As the result, LDG, the cancer predisposition gene set curated by the Washington University Genome Center, has the highest consistency score of 0.46, followed by BVG, CCG, GGG, NBG, ELG and NRG, with consistency scores of 0.19, 0.12, 0.033, 0.028, 0.00034 and 0.000064, respectively (Figs 3 and 4). This is in agreement with the results for the intersections across 5 or 6 sets. Among all the intersections across 6 sets, the one with 34 genes for the combination without NRG is the most significant (FE = 1.2 × 10^9^ and adjusted *P* value = 3.0 × 10^−302^) while the one with 59 genes for the combination without ELG and NRG is the most significant (FE = 1.1 × 10^7^ and adjusted *P* value < 1 × 10^−310^) among all the intersections across 5 sets. As NRG is the only cancer predisposition gene set with pure germline mutations and there is a very limited number of mutation genes shared between germline and soma, it is not surprising that NRG is least consistent with the other sets. The overlap between the germline cancer predisposition gene set and the somatic driver sets suggests that a majority of the intersection genes are gatekeepers maintaining cell survival. For example, the intersection of BVG, CCG and NRG includes 36 genes (Table S1), of which 30 genes are gatekeepers but only 6 are caretakers for maintaining genome integrity^24^. Collectively, our analysis provides some interesting insights into the relationships among the cancer gene census sets, which is otherwise unavailable by simply comparing intersection sizes.

### Relations between GWAS based complex phenotype susceptible gene sets

GWAS have been widely employed for identifying candidate genetic variants susceptible to complex human common diseases, including diabetes, obesity and cardiovascular disorders. As of July 23, 2014, the GWAS catalog^25^ had curated a database of 13,564 single nucleotide polymorphisms (SNPs) which were found to be associated with 1,111 complex traits from 1,937 large scale association studies. As the predicted SNPs may be generally not the true causal variants for a phenotype, but are in linkage disequilibrium (LD) with one or more causal variants^26^, the genes closest to the predicted SNPs were annotated as the candidate causal genes (called the mapped genes) by the GWAS Catalog, resulting in a total of 9,296 mapped genes in the database. It is well-recognized that there are genetic connections between distinct complex diseases or phenotypes but yet it remains challenging to quantify such connections under the framework of genotype-phenotype map. In order to examine the shared genetic component of complex traits, we used the package *SuperExactTest* to exploit the top six complex trait phenotypes which presented the most number of candidate genes according to the GWAS Catalog, including neurological diseases (NEU), inflammatory diseases (INF), cardiovascular diseases (CVD), height (HT), IgG glycosylation (IgG), as well as obesity (OB) (Table S2). The sizes of these GWAS gene sets varied from 425 (HT) to 1,207 (OB).

Figures S1A and S1B show the intersections among the six GWAS gene sets in either a circular layout or a matrix layout plotted by the package *SuperExactTest*. Again, we assume the GWAS gene sets were randomly sampled from the population of 20,687 human genes. It is interesting to note that all possible intersections were observed among the six sets associated with distinct phenotypes. Of all the intersections, the one between NEU and OB was the most significant (adjusted *P* value 1.22 × 10^−24^, FE = 2.4) (Figures S1A and S1B and Table S2). Eating behavior is neurologically associated, which appears to be an early predisposition of neurological disorder^27^. Imbalanced energy expenditure due to over-nutrition is linked to obesity, which could complicate neurological disorders. The genes shared by NEU and OB are enriched with a key pathway of axon guidance mediated by semaphorins, in which *SEMA3A* repels axons from the dorsal root ganglia, facial nerves, vagal nerves, olfactory-sensory, cortical nerves, hippocampal nerves and cerebellar nerves^28^. Significant overlap was also observed between NEU and CVD (adjusted *P* value = 2.05 × 10^−5^, FE = 1.9). The implication of nervous system related functional pathways in CVD has been confirmed by the recent integrative network approach^29^,^30^, illustrating the power of a simple intersection test in revealing the mechanistic connections upon public databases. The six sets share only one gene *TRNAI25*, transfer RNA (tRNA) isoleucine 25 (adjusted *P* value = 0.002, FE = 25081). tRNAs are the essential components in biological synthesis of new proteins and have been shown to play a crucial role in complex traits such as neurodegenerative diseases^31^,^32^. The heat-map in Fig. S1C shows the pairwise similarities among the six GWAS gene sets. The pairwise relationships between the six sets can be also depicted by a network where node size represents set size and edge width and edge color intensity are weighted by intersection size and P value significance, respectively (Fig. S1D). As expected, HT had the weakest overlap with other GWAS gene sets. Both NEU and OB are significantly overlapped with IgG (FE > 2.5 and adjusted *P* value < 2.99 × 10^−11^). Immunoglobulin G plays a critical role in chronic inflammatory processes. Many diseases are associated with inflammation (including obesity and neurological disorders), underscoring the etiological role of inflammation^23^,^33^,^34^,^35^,^36^. The results implicate the inflammatory etiology of the complex diseases that have shared genetic determinants.

## Discussion

While the methods for testing the significance of intersection between two sets have long been well established^5^,^6^, statistical test for multi-set intersections, which is even more essential to many scientific studies, has received little attention. In this paper, we presented a novel theoretical framework for efficient computation of probability distributions of multi-set intersections. This novel approach provides an axiomatic solution to perform exact statistical test of the significance of intersections by leveraging combinatorial principles. Essentially, this approach assumes the sets are comprised of independent random samples recruited from a population. The probability distribution of an intersection can be calculated through enumeration of all possible set configurations. Unlike heuristic approaches such as Poisson approximation, which require a population size to be much larger than set sizes^37^, the present approach is effective for any population and set sizes and hence is feasible for a wide range of applications. It must be stressed that enumeration of all possible set configurations is computationally intensive due to the exponential increase of the number of combinations when the number of sets is large. To reduce the computation burden, we proposed to use a forward algorithm to integrate the set configurations in a hierarchical manner, which makes the approach computationally efficient and scalable.

One big challenge in computing the probability distributions of multi-set intersection is to deal with the computational precision for very small intersection sizes and very large set sizes. As indicated in equations (4) and (5), calculation of the probability distributions of multi-set intersections requires complicated integration of hypergeometric distribution densities, in which computing binomial coefficients is the most frequent and crucial operation. When set sizes are big, numerical overflow becomes a critical issue due to large factorial numbers in calculating binomial coefficient terms of the hypergeometric function. One solution is to approximate hypergeometric distribution by binomial distribution when a population is large with respect to set sizes, such as the dhyper function in R. In the current implementation of our exact test algorithm, we employed log transformation to calculate binomial coefficient terms and this technique can increase computation efficiency and alleviate the numerical overflow issue for a vast majority of the scientific applications. Neither is perfect in terms of computation precision. However, we believe the 64-bit floating-point arithmetic, as we currently implemented within the popularly used R environment, is sufficiently accurate for a vast majority of scientific applications. To assess how the error propagation features with our package, we compared multi-set intersection probability values computed from implementation using 64-bit floating-point arithmetic with those from implementation using 32-bit floating-point arithmetic. In this analysis, we implemented the programs by C programming language. As 32-bit arithmetic is expected to be less accurate, it could lead to large difference from the more accurate 64-bit arithmetic if errors propage badly. As illustrated in the Fig. S2, however, the difference in the computed probability values between the two arithmetics is no more than three thousandths of that from 64-bit arithmetic in these examples, suggesting error propagation is well controlled. This assures a reasonably good numeric precision of the current package as implemented by 64-bit floating-point arithmetic in the R environment. While exalted levels of numeric precision is necessary in practical research or engineering, advanced programming techniques including symbolic computation and arbitrary-precision arithmetic can be readily applied to the present package.

The main interest of the present paper is in developing a statistical model for the test of significance of the overlap among *n* sets (n ≥ 3). In our model, the sizes of lower order overlaps among the sets are not of interest and hence their values compatible with the highest order overlap size will be enumerated and integrated out from the probability calculation. The present model is different from the tests of contingency tables, e.g. Pearson chi-square test, of which the primary focus is to test for relationships between several discrete variables, i.e., whether the levels of one variable are differentially distributed over the combination of levels of the other variables due to interactions among different variables^38^,^39^,^40^. All observed cell counts in a contingency table will be used in computing the hypothesis test statistics. In real data analyses, researchers may also like to test for the significance of overlaps among several sets instead of overlap among all *n* sets. To assist with a comprehensive test and presentation of all possible set combinations in the overlap analysis, we have designed a function in the *SuperExactTest* package to count and evaluate the statistical significance of all possible 2^*n*−1^ overlap combinations for *n* sets as exemplified in the applications (Fig. 1 and S1). As multiple hypothesis tests are performed, multiple testing procedures like Bonferroni correction and false discovery rate (FDR) estimation need to be employed to correct for the occurrence of false positives^41^.

To facilitate the effective visualization of multi-set intersections, we proposed two simple presentation techniques, a circular plot (Fig. 1) and a matrix plot (Fig. 2). In both plots, the configurations of multi-set intersections are plotted in a well-organized array of blocks with binary states (“presence” or “absence”) such that the intersections are independent of each other, circumventing the geometric restriction in the Venn or Euler diagram. Moreover, intersection size is represented by a bar diagram, with the statistical significance (P value) represented by the color intensity of a bar. The visualization methods are integrated with the results from the statistical test of multi-set intersections in an R package *SuperExactTest*. Users have the flexibility to change the configuration of plots, such as using different color schemes or sorting intersections by P value, set, size or degree.

Heat-map (eg, Fig. 3) is perhaps the most widely used technique to present pairwise relationships between multiple sets^42^. Alternatively, we can utilize networks to provide a more intuitive presentation of pair-wise intersections^43^,^44^. Figure 4 illustrates pairwise intersections between the gene sets in a network, with the edge width and color intensity representing the intersection size and P value significance, respectively. In the network presentation, more features (such as set size) can be readily encoded in node and edge properties like shape, size and color. Therefore, the network presentation is more advantageous to the heat-map in many instances.

We demonstrated the utility of *SuperExactTest* in two biological studies. The first one composed of seven cancer predisposition gene sets, NRG^18^, LDG^19^, GGG^17^, ELG^20^, CCG^16^, BVG^21^ and NBG^22^. These cancer predisposition gene sets represented the current understanding of the genetic risk factors in cancer. To our knowledge, the present analysis is the first comprehensive comparison of existing cancer predisposition gene collections. It is intriguing that only nine genes were shared among all seven sets though all intersections are over-represented and significant (adjusted *P* values < 2.13 × 10^−18^, FE > 11), highlighting the discrepancy in gene selection by independent researchers/groups and the limited comprehension about the cancer etiology at the current stage. To provide readers with a practical guideline on choosing the best cancer predisposition gene set in regard to the mutual consensus, we sorted the gene sets with a simple discriminative metric by integrating statistical tests of all pairwise intersections. LDG was ranked the best as it had consistently the highest consistency with other sets.

We also carried out a thorough statistical test of the intersections among six GWAS gene sets associated with complex human diseases or traits. One caveat with GWAS based gene mapping is that the predicted SNPs are generally not the true causal variants for a phenotype under study, but in LD that may harbor true causal genes. In practice, such as in the GWAS Catalog^25^, the genes adjacent to the SNPs are often considered as the most probable candidates. While there is no feasible approach to systematically evaluate every candidate, we performed a simple intersection analysis of six GWAS gene sets to check whether the inferred candidates were random genes. Of all the possible intersections among the six GWAS gene sets, those among OB, NEU and IgG showed the most significant over-representation (adjusted *P* value < 2.99 × 10^−11^), which is consistent with the comprehension that obesity complicates neurological disorders, and that both OB and NEU are associated with inflammation process, in which Immunoglobulin G plays a critical role^35^. While the polygenic feature of complex traits is notoriously difficult to dissect, the results show an interesting connection between inflammation related diseases (OB and NEU) and the immune response molecules (immunoglobulins).

In summary, this study systematically resolves several long-standing key issues regarding theory, implementation and visualization in multi-set intersection analysis. An R software package, *SuperExactTest*, was accordingly developed to unify the statistical testing and visualization of multi-set intersections. For the first time, we are able to assess the significance of intersections among a large number of sets derived from a large population. Applications of *SuperExactTest* to multiple gene sets associated with complex human diseases or traits revealed novel insights into high-order relationships among these gene sets, which are otherwise unavailable through the traditional pair-wise Fisher’s exact test. Integration of *SuperExactTest* with other systems or statistical analyses will yield more in-depth understanding of interactions or connections among multiple components involved in complex systems. We expect *SuperExactTest* developed by this study will have a broad range of applications in many disciplines such as biological sciences, social sciences, engineering, physics and economics.

## Methods

### Fold Enrichment

Fold enrichment (FE) is used to evaluate whether there is an overrepresentation or underrepresentation of an intersection relative to the random expectation. Given a population of elements *P* and its two subsets, *A* and *B*, which share a collection of elements 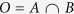, FE (also known as fold change in some literatures) is defined as the ratio between the observed and expected fractions of intersection (or overlap) elements:




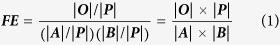




where, |*X*| denotes the number of elements in a set *X*. The standard Fisher’s exact test (FET) or hypergeometric test can be employed to calculate the statistical significance of the observed overlap^7^. The above equation can be readily generalized as below to analyze the overlap among *N* ( > 2) sets, 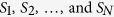:




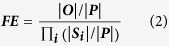




### Exact Test of Multi-Set Intersections

Here, we developed a novel algorithm for fast calculation of the exact probability distributions of multi-set intersections. This algorithm is scalable to any number of sets.

Let’s first consider a population *P* of *n* elements and its two subsets *A* and *B* with *a* and *b* elements, respectively. Assume that all the elements in *P* are unbiased, i.e., they are equally likely to be sampled into each set. The probability of observing *x* elements shared by *A* and *B* can be calculated by combinatorial principles:




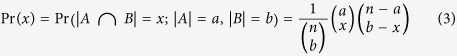




**Figure 5:**
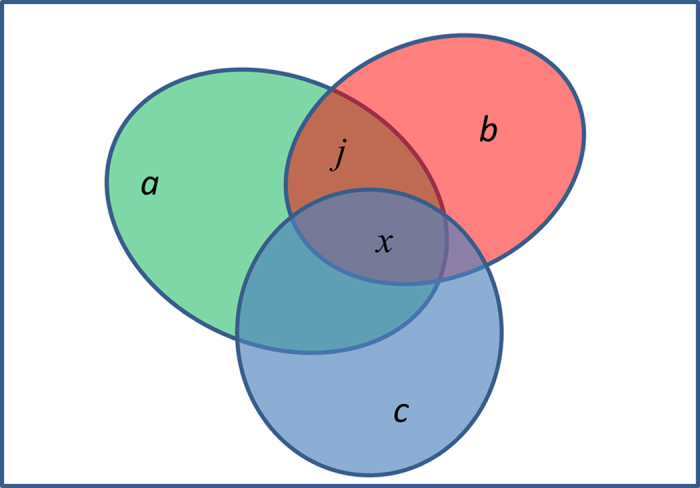
A Venn diagram showing intersection among three sets. The box represents a population P of *n* elements and the three ellipses are three subsets *A*, *B* and *C*, with size *a*, *b* and *c*, respectively. *A* and *B* share *j* elements, among which *x* elements are also shared with *C*.

Note that this equation is exactly the same as the density function of a hypergeometric model that samples *x* white balls in *b* draw without replacement from an urn of *a* white balls and *n*-*a* black balls^6^. In R, the hypergeometric density value can be calculated by the function *dhyper*(*x*, *a*, *n*-*a*, *b*). Summing up the density values from *x* to maximum possible overlap size gives rise to the one-tailed probability that the cardinality of the intersection equals to or exceeds *x*. In cases that there are three sets *A*, *B* and *C*, with *a*, *b* and *c* elements, respectively (as exemplified in Fig. 5), the probability that the three sets share *x* common elements can be calculated by enumerating possible intersections in a hierarchical way:




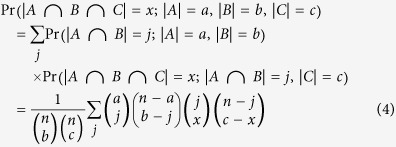




In the equation (4), the sum integrates over all possible values of *j*, i.e., all possible intersections between sets *A* and *B* which are compatible with the observed number of intersections between all the three sets. Note that the equation (4) is essentially a sum of products of hypergeometric densities. One caveat of computing the equation (4) is that a direct calculation of those binomial coefficients results in numeric overflow for large sets.

The equation (4) can be readily extended to test for overlaps among four or more sets. For example, in the case of five sets *A*, *B*, *C*, *D* and *E* with *a*, *b*, *c*, *d* and *e* elements, respectively, the probability of sharing *x* elements among them can be determined by




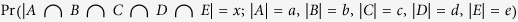







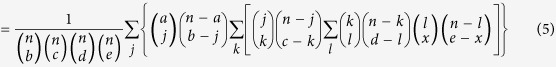




where, the sum traverses all possible intersections of size *j* between *A* and *B*, each of which is further intersected with *C* by size *k*, later intersected with *D* by size *l*, until finally intersected with the last set *E*. The order of the sets in the hierarchical intersections does not affect the outcome.

### Efficient Calculation of the Exact Probability of Multi-Set Intersection

As shown in the equations (4) and (5), the calculation of the probabilities of multi-set intersections involves integrations over all possible hierarchical intersections across all the sets. When the population size and/or the number of sets increase, the number of operations in a naïve integration approach increases exponentially, making it impractical to calculate probabilities in a reasonable time. To optimize the procedure described in the equation (5), we developed a forward algorithm which was originally developed to calculate hidden Markov models^45^.

For simplicity without loss of generality, let’s consider an example of 5 sets with *a*, *b*, *c*, *d* and *e* elements, respectively. As indicated in the equation (5), the inner-most summation over *l* is a function of *k*, denoted by 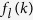, which, given *k*, is independent of variables *a*, *b* and *c*, and *j*. Therefore we can calculate 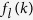 with respect to all possible *k* values without considering the actual values of variables *a*, *b*, *c* and *j*. Analogously, we can treat the second inner-most summation as a function of *j*, say 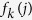, which is independent of variables *a* and *b* given *j*. By utilizing the pre-computed function values of 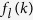, 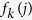 can be quickly computed without going through the integration of the inner-most summation for each *j*. Finally the outer-most summation can be done efficiently after calculating 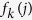 for all possible *j*. This procedure is detailed below.

Step 1. Compute the inner-most integration over *l* as a function of *k*




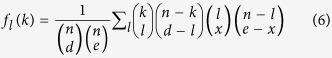




Step 2. Compute the integration over *k* as a function of *j*




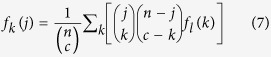




Step 3. Compute the multi-set intersection probability from the outer-most integration




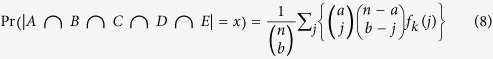




The computation complexity of this procedure is estimated as follows. Given a population of *n* elements, we consider *t* sets which share *x* common elements and the smallest set size is *m*. Equation (6) is essentially a sum of a product of two hypergeometric density values. The loop variable *l* takes a value from *x* to *k*, where *k* takes a value from *x* to the smallest set size *m*. The number of operations in Step 1 is (m − x) * (m − x + 1)/2 ≈ (m^2)/2 when x ≪ m. Therefore, the worst computation complexity for Step 1 is *O(m*^*2*^). Step 2 has the same computation complexity as Step 1. The computation time in Equation (8) is linear to *j* (*j* = *x, x* + *1*,.., *m*), so the worst computation complexity of Step 3 is *O(m)*. In total, we need to run Step 1 by once, Step 2 by *(t* *−* *3)* times, and Step 3 by only once. Therefore the overall worst computation complexity for the whole procedure is *O(t* * *m*^*2*^). When 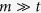, the complexity will be 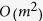. Since the computation complexity of the algorithm is linear with respect to the number of sets for intersection test, it is feasible to compute intersection probabilities for a large number of sets.

### Software Availability

SuperExactTest is available as R package in CRAN (the Comprehensive R Archive Network, https://cran.r-project.org/), a repository of open-source software.

Below is the R code for using the *SuperExactTest* package to analyze the cancer gene sets.

Step 1. Load the *SuperExactTest* package. > *library(SuperExactTest)*

Step 2. Read in the cancer gene. > *data(Cancer)*

Step 3. Check the cancer data object. > *str(Cancer)*

Step 4. Perform the super exact test. > *Result1 = supertest(Cancer, n* = *20687)*

Step 5. Visualize the result in a circular layout. > *plot(Result1, degree* = *2:7, sort.by = ‘size’, legend.col *= *1)*

Step 6. Visualize the result in a matrix layout. > *plot(Result1, Layout* = *‘landscape’, degree* = *2:7, sort.by* = *‘size’)*

Step 7. Tabulate the analysis result into a file: > *write.csv(summary(Result1)$Table, file* = *‘summary.table.csv’, row.names* = *FALSE)*

For more detailed explanation regarding the package usage, please read R help documents > *help(package* = *‘SuperExactTest’)*.

## Additional Information

**How to cite this article**: Wang, M. *et al.* Efficient Test and Visualization of Multi-Set Intersections. *Sci. Rep.* **5**, 16923; doi: 10.1038/srep16923 (2015).

## Supplementary Material

Supplementary Information

## Figures and Tables

**Table 1:** Parameters defining weighted sampling and empirical false positive rate of the present method for computing significance of overlap among three sets from weighted sampling.

Weight(*w*)	Population Size (*n*)									
	1000	2000	3000	4000	5000	6000	7000	8000	9000	10000
1.0	0.036	0.04	0.01	0.009	0.018	0.026	0.01	0.005	0.033	0.024
1.1	0.05	0.035	0.014	0.018	0.015	0.039	0.015	0.01	0.038	0.026
1.2	0.054	0.035	0.023	0.018	0.015	0.038	0.014	0.004	0.03	0.02
1.3	0.052	0.035	0.018	0.024	0.018	0.025	0.017	0.006	0.04	0.021
1.4	0.067	0.053	0.024	0.02	0.013	0.028	0.007	0.006	0.042	0.023
1.5	0.078	0.04	0.021	0.014	0.019	0.027	0.011	0.01	0.036	0.023
1.6	0.084	0.047	0.022	0.02	0.022	0.029	0.012	0.003	0.037	0.024
1.7	0.102	0.062	0.015	0.019	0.013	0.029	0.008	0.009	0.03	0.033
1.8	0.137	0.057	0.029	0.027	0.017	0.033	0.014	0.007	0.041	0.024
1.9	0.157	0.063	0.03	0.021	0.028	0.029	0.013	0.008	0.038	0.031
2.0	0.178	0.078	0.035	0.029	0.022	0.041	0.014	0.008	0.028	0.027

The rate of false positive was calculated as the fraction of simulations with P value < 0.05 in 1000 repeated simulations. In each simulation, we sampled independently three sets of sizes 200, 300 and 400, from a population of size *n*. In each population, 100 elements had a sampling probability weight of *w* over the rest of the elements: all elements in the population were equally likely to be sampled (i.e. unbiased sampling) if *w* = 1, while 100 of the elements had twice the chance to be sampled compared with the others if *w* = 2.

**Table 2:** Seven cancer predisposition gene sets.

Geneset	Size	Reference
BVG	125	Vogelstein, B. *et al.* Cancer genome landscapes. Science 2013, 339:1546-1558
CCG	522	Futreal, P. A. *et al.* A census of human cancer genes. Nature reviews. Cancer 2004, 4:177-183
ELG	107	Garraway, L. A. & Lander, E. S. Lessons from the cancer genome. Cell 2013, 153:17-37
GGG	260	Lawrence, M. S. *et al.* Discovery and saturation analysis of cancer genes across 21 tumour types. Nature 2014, 505:495-501
LDG	137	Kandoth, C. *et al.* Mutational landscape and significance across 12 major cancer types. Nature 2013, 502:333-339
NBG	435	Tamborero, D. *et al.* Comprehensive identification of mutational cancer driver genes across 12 tumor types. Scientific reports 2013, 3:2650
NRG	114	Rahman, N. Realizing the promise of cancer predisposition genes. Nature 2014, 505:302-308
