# Supplementary material for: Efficient Test and Visualization of Multi-Set Intersections
Source: Sci Rep. 2015 Nov 25;5:16923. doi: 10.1038/srep16923 (PMC4658477; doi:10.1038/srep16923)
Supplement: Supplementary Information [file srep16923-s1.pdf]

Efficient Test and Visualization of Multi-Set Intersections

Minghui Wang, Yongzhong Zhao and Bin Zhang*

Department of Genetics and Genomic Sciences, Icahn Institute of Genomics and Multiscale Biology, Icahn School of Medicine at Mount Sinai, 1470 Madison Avenue, NY 10029, USA.

*Correspondence:
Bin Zhang, PhD.
Associate Professor
Department of Genetics & Genomic Sciences
Icahn School of Medicine at Mount Sinai
1470 Madison Avenue, Room S8-111, New York, NY 10029
(Phone) 212-824-8947 (Fax) 646-537-8660
Email: bin.zhang@mssm.edu

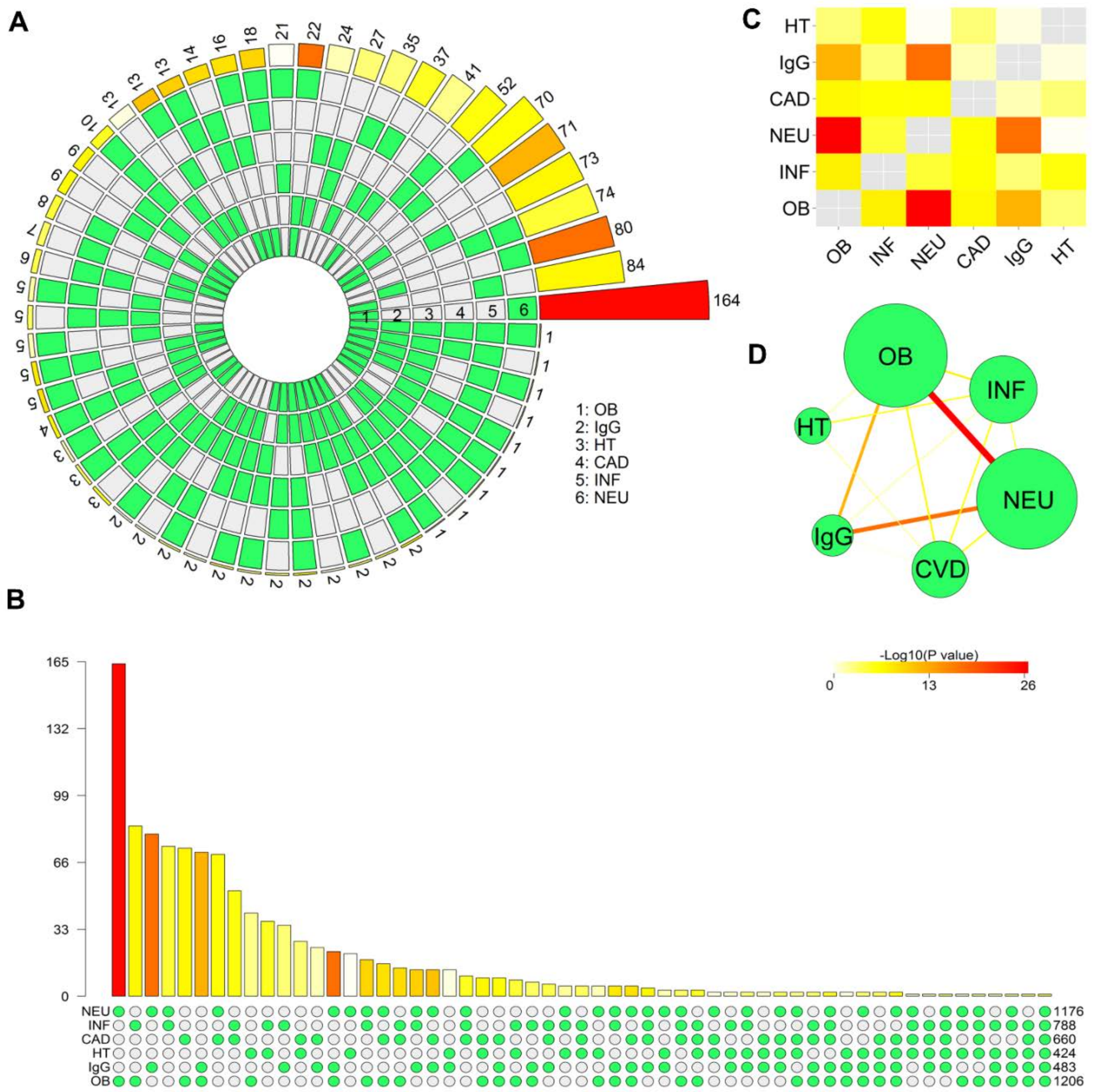

Figure S1 | Visualization of the intersections of six GWAS gene sets. **A)** and **B)** show all possible intersections among the six gene sets in a circular and matrix layouts, respectively. **C)** and **D)** show the pairwise intersections. The node size in **D)** is proportional to the gene set size. The rest of the legend is identical to that of **Figures 1-4**.

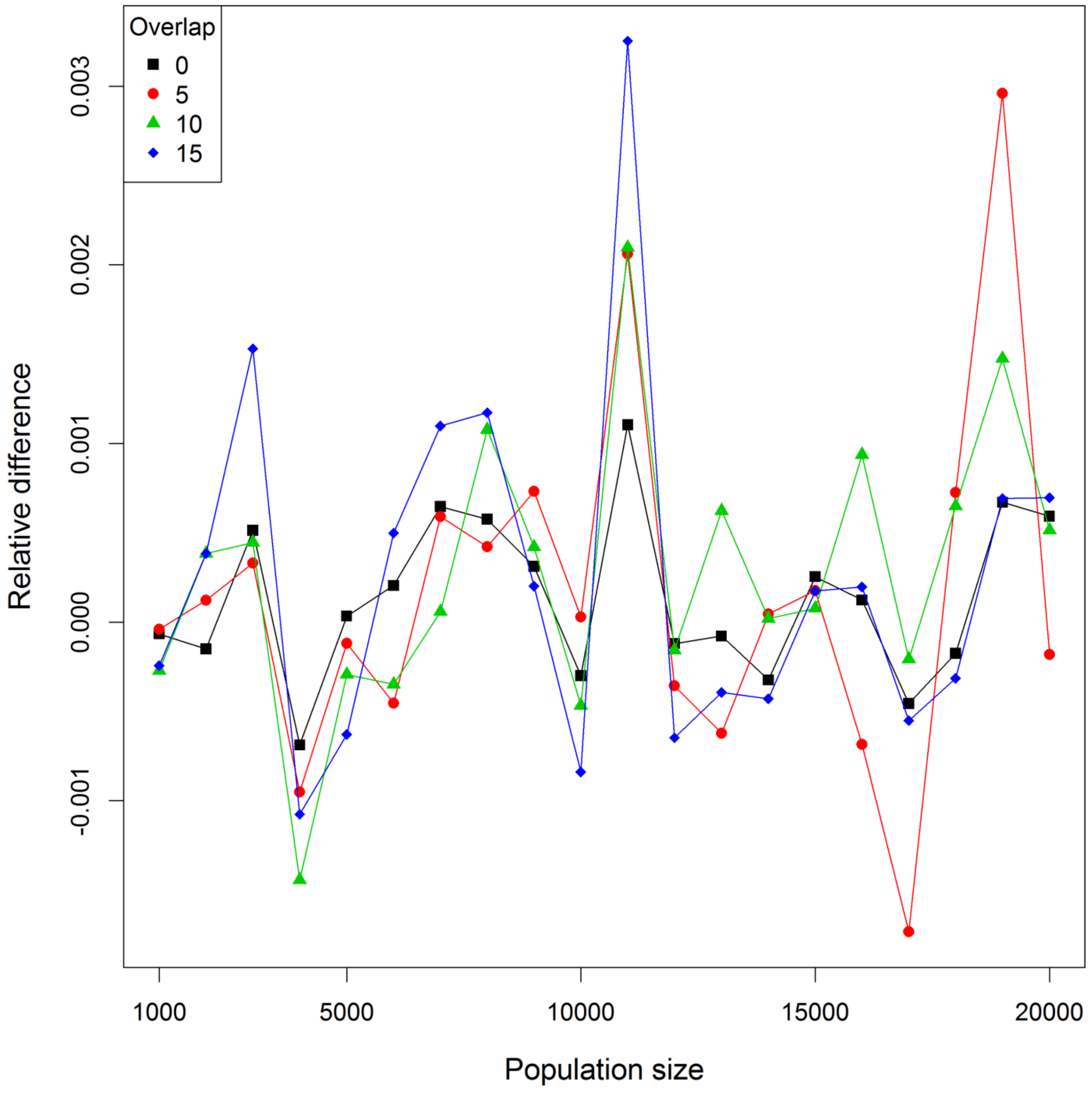

Figure S2 | Comparing the multi-set intersection probability density values between implementations using single-precision (32-bit) and double-precision (64-bit) floating-point arithmetics in C language. At each point, the intersection probabilities are computed for an overlap of size 0, 5, 10 and 15 among three hypothetical sets of sizes 200, 300 and 400, which are randomly sampled from a population. The y axis is the difference between the probabilities

obtained from the two arithmetics divided by the probability computed from the double-precision arithmetic.

Table S1. Intersection analysis of cancer gene sets.

Intersections	Observed Intersection Size	Expected Intersection Size	Fold Enrichment	P value	Adjusted P value*
NRG[1]	114	NA	NA	NA	NA
NBG[2]	435	NA	NA	NA	NA
LDG[3]	137	NA	NA	NA	NA
GGG[4]	260	NA	NA	NA	NA
ELG[5]	107	NA	NA	NA	NA
CCG[6]	522	NA	NA	NA	NA
BVG[7]	125	NA	NA	NA	NA
NBG & NRG	41	2.40	17.10	1.10E-39	1.32E-37
LDG & NRG	38	0.75	50.33	1.01E-55	1.21E-53
LDG & NBG	83	2.88	28.81	3.71E-105	4.45E-103
GGG & NRG	31	1.43	21.64	6.06E-33	7.28E-31
GGG & NBG	120	5.47	21.95	4.91E-134	5.89E-132
GGG & LDG	80	1.72	46.46	1.30E-119	1.56E-117
ELG & NRG	17	0.59	28.83	1.78E-20	2.13E-18
ELG & NBG	53	2.25	23.56	2.32E-60	2.79E-58
ELG & LDG	52	0.71	73.38	6.97E-88	8.37E-86
ELG & GGG	54	1.34	40.15	4.84E-75	5.81E-73
CCG & NRG	76	2.88	26.42	1.48E-94	1.78E-92
CCG & NBG	129	10.98	11.75	2.62E-103	3.14E-101
CCG & LDG	119	3.46	34.42	4.31E-175	5.18E-173
CCG & GGG	94	6.56	14.33	1.23E-83	1.48E-81
CCG & ELG	63	2.70	23.33	4.12E-73	4.94E-71
BVG & NRG	38	0.69	55.17	1.76E-57	2.11E-55
BVG & NBG	78	2.63	29.68	3.18E-100	3.82E-98
BVG & LDG	119	0.83	143.75	1.39E-285	1.67E-283
BVG & GGG	76	1.57	48.38	2.13E-115	2.56E-113
BVG & ELG	48	0.65	74.24	5.52E-81	6.62E-79
BVG & CCG	110	3.15	34.87	6.19E-163	7.42E-161
LDG & NBG & NRG	25	0.02	1574.78	1.92E-73	2.30E-71
GGG & NBG & NRG	26	0.03	862.98	4.15E-69	4.98E-67
GGG & LDG & NRG	25	0.01	2634.73	3.05E-79	3.66E-77
GGG & LDG & NBG	69	0.04	1905.73	3.13E-214	3.76E-212
ELG & NBG & NRG	13	0.01	1048.48	5.04E-36	6.05E-34
ELG & LDG & NRG	14	0.00	3585.20	1.99E-46	2.39E-44
ELG & LDG & NBG	43	0.01	2885.82	1.89E-140	2.27E-138
ELG & GGG & NRG	13	0.01	1754.19	5.55E-39	6.66E-37
ELG & GGG & NBG	48	0.03	1697.42	9.43E-145	1.13E-142
ELG & GGG & LDG	44	0.01	4940.49	6.97E-155	8.36E-153
CCG & NBG & NRG	35	0.06	578.63	5.00E-87	6.01E-85
CCG & LDG & NRG	36	0.02	1889.74	1.26E-109	1.51E-107
CCG & LDG & NBG	73	0.07	1004.24	7.51E-205	9.02E-203
CCG & GGG & NRG	26	0.04	719.15	5.38E-67	6.46E-65

CCG & GGG & NBG	73	0.14	529.16	4.85E-179	5.82E-177
CCG & GGG & LDG	73	0.04	1680.17	3.03E-223	3.63E-221
CCG & ELG & NRG	15	0.01	1008.15	3.30E-41	3.96E-39
CCG & ELG & NBG	43	0.06	757.39	4.90E-113	5.88E-111
CCG & ELG & LDG	47	0.02	2628.56	2.85E-152	3.42E-150
CCG & ELG & GGG	45	0.03	1326.11	3.16E-130	3.79E-128
BVG & NBG & NRG	25	0.01	1725.96	1.52E-74	1.83E-72
BVG & LDG & NRG	38	0.00	8329.97	1.34E-142	1.61E-140
BVG & LDG & NBG	78	0.02	4480.95	7.09E-282	8.51E-280
BVG & GGG & NRG	25	0.01	2887.67	2.42E-80	2.91E-78
BVG & GGG & NBG	66	0.03	1997.87	1.42E-206	1.71E-204
BVG & GGG & LDG	76	0.01	7304.74	3.79E-292	4.54E-290
BVG & ELG & NRG	14	0.00	3929.38	5.16E-47	6.19E-45
BVG & ELG & NBG	41	0.01	3015.75	1.15E-134	1.38E-132
BVG & ELG & LDG	47	0.00	10976.87	7.48E-185	8.98E-183
BVG & ELG & GGG	42	0.01	5168.65	1.34E-148	1.61E-146
BVG & CCG & NRG	36	0.02	2071.15	2.70E-111	3.23E-109
BVG & CCG & NBG	69	0.07	1040.34	5.87E-195	7.04E-193
BVG & CCG & LDG	105	0.02	5026.70	0	0.00E+00
BVG & CCG & GGG	69	0.04	1740.57	3.23E-212	3.88E-210
BVG & CCG & ELG	43	0.02	2635.72	5.95E-139	7.14E-137
GGG & LDG & NBG & NRG	21	0.00	105250.41	3.00E-100	3.60E-98
ELG & LDG & NBG & NRG	11	0.00	133963.59	6.01E-54	7.22E-52
ELG & GGG & NBG & NRG	11	0.00	70588.51	8.43E-51	1.01E-48
ELG & GGG & LDG & NRG	12	0.00	244506.97	5.61E-62	6.73E-60
ELG & GGG & LDG & NBG	40	0.00	213592.30	2.87E-206	3.45E-204
CCG & LDG & NBG & NRG	24	0.00	59912.66	1.30E-108	1.56E-106
CCG & GGG & NBG & NRG	21	0.00	27623.19	1.59E-87	1.91E-85
CCG & GGG & LDG & NRG	24	0.00	100238.49	3.58E-114	4.30E-112
CCG & GGG & LDG & NBG	62	0.00	67862.61	8.96E-289	1.08E-286
CCG & ELG & NBG & NRG	11	0.00	35159.03	2.01E-47	2.41E-45
CCG & ELG & LDG & NRG	13	0.00	131933.84	1.47E-63	1.77E-61
CCG & ELG & LDG & NBG	38	0.00	101067.62	1.36E-182	1.63E-180
CCG & ELG & GGG & NRG	11	0.00	58823.76	6.40E-50	7.68E-48
CCG & ELG & GGG & NBG	40	0.00	56057.75	6.05E-181	7.26E-179
CCG & ELG & GGG & LDG	40	0.00	177993.58	5.79E-203	6.95E-201
BVG & LDG & NBG & NRG	25	0.00	260620.07	5.07E-130	6.08E-128
BVG & GGG & NBG & NRG	21	0.00	115354.45	3.71E-101	4.45E-99
BVG & GGG & LDG & NRG	25	0.00	436037.43	8.03E-136	9.64E-134
BVG & GGG & LDG & NBG	66	0.00	301677.76	0	0.00E+00
BVG & ELG & NBG & NRG	11	0.00	146824.09	2.11E-54	2.53E-52
BVG & ELG & LDG & NRG	14	0.00	593336.92	8.15E-78	9.78E-76
BVG & ELG & LDG & NBG	41	0.00	455378.78	6.92E-227	8.30E-225
BVG & ELG & GGG & NRG	12	0.00	267979.64	1.78E-62	2.13E-60
BVG & ELG & GGG & NBG	38	0.00	222392.30	1.64E-196	1.97E-194
BVG & ELG & GGG & LDG	42	0.00	780466.25	3.74E-243	4.49E-241
BVG & CCG & NBG & NRG	24	0.00	65664.28	1.15E-109	1.39E-107

BVG & CCG & LDG & NRG	36	0.00	312744.09	6.42E-192	7.71E-190
BVG & CCG & LDG & NBG	69	0.00	157091.00	0	0.00E+00
BVG & CCG & GGG & NRG	24	0.00	109861.38	3.18E-115	3.82E-113
BVG & CCG & GGG & NBG	59	0.00	70778.51	5.65E-276	6.79E-274
BVG & CCG & GGG & LDG	69	0.00	262825.32	0	0.00E+00
BVG & CCG & ELG & NRG	13	0.00	144599.49	4.22E-64	5.07E-62
BVG & CCG & ELG & NBG	36	0.00	104940.10	1.69E-173	2.03E-171
BVG & CCG & ELG & LDG	42	0.00	388737.98	1.18E-229	1.42E-227
BVG & CCG & ELG & GGG	38	0.00	185326.92	2.23E-193	2.68E-191
ELG & GGG & LDG & NBG & NRG	10	0.00	9689876.83	8.89E-68	1.07E-65
CCG & GGG & LDG & NBG & NRG	20	0.00	3972478.24	3.88E-127	4.66E-125
CCG & ELG & LDG & NBG & NRG	10	0.00	4826375.43	1.03E-64	1.24E-62
CCG & ELG & GGG & NBG & NRG	9	0.00	2288815.73	2.38E-55	2.86E-53
CCG & ELG & GGG & LDG & NRG	11	0.00	8882387.09	4.57E-74	5.48E-72
CCG & ELG & GGG & LDG & NBG	36	0.00	7618247.99	2.40E-241	2.88E-239
BVG & GGG & LDG & NBG & NRG	21	0.00	17418522.59	1.30E-147	1.56E-145
BVG & ELG & LDG & NBG & NRG	11	0.00	22170438.19	1.50E-78	1.80E-76
BVG & ELG & GGG & NBG & NRG	10	0.00	10620105.01	3.44E-68	4.12E-66
BVG & ELG & GGG & LDG & NRG	12	0.00	40464925.64	7.73E-89	9.28E-87
BVG & ELG & GGG & LDG & NBG	38	0.00	33581237.14	9.21E-282	1.11E-279
BVG & CCG & LDG & NBG & NRG	24	0.00	9915305.69	6.97E-163	8.36E-161
BVG & CCG & GGG & NBG & NRG	20	0.00	4353836.15	5.35E-128	6.42E-126
BVG & CCG & GGG & LDG & NRG	24	0.00	16589069.13	1.92E-168	2.31E-166
BVG & CCG & GGG & LDG & NBG	59	0.00	10687555.46	0	0.00E+00
BVG & CCG & ELG & NBG & NRG	10	0.00	5289707.47	4.00E-65	4.79E-63
BVG & CCG & ELG & LDG & NRG	13	0.00	21834522.46	1.11E-92	1.33E-90
BVG & CCG & ELG & LDG & NBG	36	0.00	15845955.82	4.00E-254	4.80E-252
BVG & CCG & ELG & GGG & NRG	11	0.00	9735096.26	1.60E-74	1.92E-72
BVG & CCG & ELG & GGG & NBG	34	0.00	7885733.14	2.57E-228	3.09E-226
BVG & CCG & ELG & GGG & LDG	38	0.00	27984364.29	1.25E-278	1.50E-276
CCG & ELG & GGG & LDG & NBG & NRG	9	0.00	345611175.84	4.47E-75	5.37E-73
BVG & ELG & GGG & LDG & NBG & NRG	10	0.00	1603635855.91	3.99E-90	4.79E-88
BVG & CCG & GGG & LDG & NBG & NRG	20	0.00	657429258.94	3.31E-172	3.97E-170
BVG & CCG & ELG & LDG & NBG & NRG	10	0.00	798745828.61	4.64E-87	5.57E-85
BVG & CCG & ELG & GGG & NBG & NRG	9	0.00	378789848.72	1.91E-75	2.29E-73
BVG & CCG & ELG & GGG & LDG & NRG	11	0.00	1469999534.59	1.14E-98	1.37E-96
BVG & CCG & ELG & GGG & LDG & NBG	34	0.00	1190745704.50	2.47E-304	2.97E-302
BVG & CCG & ELG & GGG & LDG & NBG & NRG	9	0.00	#############	3.58E-95	4.30E-93

1, NRG (Rahman, N. Realizing the promise of cancer predisposition genes. Nature 2014, 505:302-308)

2, NBG (Tamborero, D. et al. Comprehensive identification of mutational cancer driver genes across 12 tumor types. Scientific reports 2013, 3:2650).

3, LDG (Kandoth, C. et al. Mutational landscape and significance across 12 major cancer types. Nature 2013, 502:333-339)

4, GGG (Lawrence, M. S. et al. Discovery and saturation analysis of cancer genes across 21 tumour types. Nature 2014, 505:495-501)

5, ELG (Garraway, L. A. & Lander, E. S. Lessons from the cancer genome. Cell 2013, 153:17-37)

6, CCG (Futreal, P. A. et al. A census of human cancer genes. Nature reviews. Cancer 2004, 4:177-183)

7, BVG (Vogelstein, B. et al. Cancer genome landscapes. Science 2013, 339:1546-1558)

*Bonferroni corrected P value

Table S2. Intersection analysis of GWAS gene sets.

Intersections	Observed Intersection Size	Expected Intersection Size	Fold Enrichment	P value	Adjusted P value*
NEU[1]	1176	NA	NA	NA	NA
INF[2]	788	NA	NA	NA	NA
CVD[3]	660	NA	NA	NA	NA
HT[4]	424	NA	NA	NA	NA
IgG[5]	483	NA	NA	NA	NA
OB[6]	1206	NA	NA	NA	NA
INF & NEU	74	44.80	1.65	1.48E-05	8.43E-04
CVD & NEU	70	37.52	1.87	3.59E-07	2.05E-05
CVD & INF	52	25.14	2.07	6.39E-07	3.64E-05
HT & NEU	21	24.10	0.87	0.77	1.00
HT & INF	37	16.15	2.29	2.68E-06	1.53E-04
HT & CVD	27	13.53	2.00	5.61E-04	0.03
IgG & NEU	80	27.46	2.91	3.81E-18	2.17E-16
IgG & INF	35	18.40	1.90	2.25E-04	0.01
IgG & CVD	24	15.41	1.56	0.02	1.00
IgG & HT	13	9.90	1.31	0.19	1.00
OB & NEU	164	68.56	2.39	2.15E-26	1.22E-24
OB & INF	84	45.94	1.83	5.76E-08	3.28E-06
OB & CVD	73	38.48	1.90	1.02E-07	5.84E-06
OB & HT	41	24.72	1.66	1.05E-03	0.06
OB & IgG	71	28.16	2.52	5.25E-13	2.99E-11
CVD & INF & NEU	10	1.43	7.00	2.39E-06	1.36E-04
HT & INF & NEU	5	0.92	5.45	2.48E-03	0.14
HT & CVD & NEU	2	0.77	2.60	0.18	1.00
HT & CVD & INF	5	0.52	9.70	1.90E-04	1.08E-02
IgG & INF & NEU	13	1.05	12.43	8.32E-11	4.74E-09
IgG & CVD & NEU	13	0.88	14.84	9.48E-12	5.40E-10
IgG & CVD & INF	6	0.59	10.22	3.25E-05	1.85E-03
IgG & HT & NEU	3	0.56	5.33	0.02	1.00
IgG & HT & INF	2	0.38	5.30	0.06	1.00
IgG & HT & CVD	2	0.32	6.33	0.04	1.00
OB & INF & NEU	18	2.61	6.89	3.07E-10	1.75E-08
OB & CVD & NEU	16	2.19	7.32	1.27E-09	7.27E-08
OB & CVD & INF	14	1.47	9.55	4.78E-10	2.72E-08
OB & HT & NEU	5	1.41	3.56	0.01	0.81
OB & HT & INF	8	0.94	8.50	6.06E-06	3.45E-04
OB & HT & CVD	9	0.79	11.41	1.39E-07	7.95E-06
OB & IgG & NEU	22	1.60	13.74	2.88E-18	1.64E-16
OB & IgG & INF	7	1.07	6.53	1.20E-04	6.85E-03
OB & IgG & CVD	9	0.90	10.02	4.14E-07	2.36E-05
OB & IgG & HT	2	0.58	3.47	0.11	1.00
HT & CVD & INF & NEU	1	0.03	34.14	0.03	1.00
IgG & CVD & INF & NEU	4	0.03	119.87	4.87E-08	2.77E-06

IgG & HT & INF & NEU	2	0.02	93.30	2.25E-04	0.01
IgG & HT & CVD & NEU	2	0.02	111.39	1.58E-04	9.02E-03
IgG & HT & CVD & INF	1	0.01	83.12	0.01	0.68
OB & CVD & INF & NEU	3	0.08	36.01	8.95E-05	5.10E-03
OB & HT & INF & NEU	1	0.05	18.68	0.05	1.00
OB & HT & CVD & NEU	2	0.04	44.61	9.70E-04	5.53E-02
OB & HT & CVD & INF	3	0.03	99.87	4.34E-06	2.48E-04
OB & IgG & INF & NEU	5	0.06	82.00	6.36E-09	3.63E-07
OB & IgG & CVD & NEU	5	0.05	97.91	2.64E-09	1.50E-07
OB & IgG & CVD & INF	2	0.03	58.45	5.69E-04	0.03
OB & IgG & HT & NEU	2	0.03	60.96	5.24E-04	0.03
OB & IgG & HT & INF	1	0.02	45.49	0.02	1.00
OB & IgG & HT & CVD	2	0.02	108.62	1.66E-04	9.48E-03
IgG & HT & CVD & INF & NEU	1	6.84E-04	1462.18	6.84E-04	0.04
OB & HT & CVD & INF & NEU	1	1.71E-03	585.60	1.71E-03	9.73E-02
OB & IgG & CVD & INF & NEU	2	1.95E-03	1028.13	1.88E-06	1.07E-04
OB & IgG & HT & INF & NEU	1	1.25E-03	800.20	1.25E-03	0.07
OB & IgG & HT & CVD & NEU	2	1.05E-03	1910.77	5.43E-07	3.10E-05
OB & IgG & HT & CVD & INF	1	7.01E-04	1425.80	7.01E-04	0.04
OB & IgG & HT & CVD & INF & NEU	1	3.99E-05	25081.29	3.99E-05	2.27E-03

1, NEU (Bipolar disorder and schizophrenia, Schizophrenia, Major depressive disorder, Alzheimer's disease, Parkinson's disease, Cognitive performance, Bipolar disorder);

2, INF (Crohn's disease, Ulcerative colitis, Inflammatory bowel disease, Rheumatoid arthritis, Multiple sclerosis, Systemic lupus erythematosus);

3, CVD (Type 2 diabetes, Coronary heart disease, Blood pressure, total Cholesterol, HDL cholesterol, Triglycerides);

4, HT (height);

5, IgG (IgG glycosylation);

6, OB (obesity, obesity related traits);

*Bonferroni corrected P value
